# Hormonal staining patterns and clinical outcomes in acromegaly: a 15-year single-center study

**DOI:** 10.1007/s11102-026-01688-4

**Published:** 2026-05-18

**Authors:** Sinem Başak Tan Öksüz, Esra Eraslan Aydemir, Aylin Okçu Heper, Cevriye Cansız Ersöz, Murat Faik Erdoğan, Rıfat Emral, Mustafa Şahin, Özgür Demir, Asena Gökçay Canpolat, Sevim Güllü

**Affiliations:** 1 Department of Endocrinology and Metabolism, Ankara University School of Medicine, Ankara, Türkiye; 2 Department of Pathology, Ankara University School of Medicine, Ankara, Türkiye

**Keywords:** Acromegaly, Prolactin co-expression, Pituitary adenoma, Immunohistochemistry, Somatostatin analogue

## Abstract

**Purpose:**

Acromegaly is most commonly caused by pituitary somatotroph adenomas, a substantial proportion of which exhibit prolactin (PRL) expression on immunohistochemistry. However, the clinical relevance and prognostic implications of this pathological phenotype remain controversial. We aimed to compare pathological features and treatment outcomes of growth hormone/prolactin (GH/PRL)–positive adenomas with GH-only adenomas.

**Methods:**

We retrospectively analyzed 90 patients with acromegaly who underwent transsphenoidal surgery between 2010 and 2025. Adenomas were classified according to immunohistochemical staining as GH-only, GH/PRL, or other subtypes. Clinical, biochemical, radiological, and treatment outcomes were compared across groups.

**Results:**

Of 90 adenomas, 47 (52.2%) were GH-only, 39 (43.3%) GH/PRL, and 4 (4.4%) other PIT-1 lineage adenomas. Postoperative remission was achieved in 32 patients (35.6%), with no significant difference between GH-only and GH/PRL adenomas. In multivariable analysis, optic chiasm compression was independently associated with lower odds of remission (OR 0.212, 95% CI 0.049–0.910; *p* = 0.037). Among 45 patients receiving SSA therapy, 25 (55.6%) achieved biochemical response. Perinuclear CK8/18 staining (OR 4.839, 95% CI 1.198–19.540; *p* = 0.027) and T2-weighted MRI hypointensity (OR 5.464, 95% CI 1.284–23.250; *p* = 0.022) were independently associated with SSA response. Although Ki-67 was higher in GH/PRL adenomas, it was not associated with treatment outcomes.

**Conclusion:**

Despite higher proliferative activity, GH/PRL adenomas did not demonstrate inferior surgical or medical outcomes compared with GH-only adenomas. Prognosis appears to be driven primarily by anatomical features and cytokeratin patterns, supporting individualized treatment strategies.

## Introduction

Acromegaly is a rare disorder caused by chronic hypersecretion of growth hormone (GH) and consequent elevation of insulin-like growth factor-1 (IGF-1), leading to increased morbidity and mortality through cardiovascular, metabolic, and musculoskeletal complications [1]. The vast majority of acromegaly cases are caused by pituitary neuroendocrine tumors (PitNETs), also known as pituitary adenomas, arising from somatotroph cells. Nevertheless, variability in pathological and clinical characteristics is commonly observed across cases. Beyond purely GH-secreting adenomas, a substantial subset of patients (reported as 25–30% of cases) harbor adenomas exhibiting concomitant prolactin (PRL) expression [2, 3]. However, the true prevalence and prognostic impact of GH/PRL adenomas remain uncertain because of variable diagnostic definitions across studies and limited patient numbers.

Classical predictors of outcome include age, sex, baseline GH and IGF-1 levels, tumor size, and radiological invasiveness [1, 4–6]. More recently, immunohistochemical (IHC) features, particularly hormone expression patterns such as GH and GH/PRL co-expression, have been explored as potential indicators of tumor behavior. However, the available evidence remains inconclusive, as studies examining the relationship between IHC-defined tumor characteristics and clinical outcomes have yielded heterogeneous results. Some studies have suggested worse remission rates in GH/PRL adenomas compared with GH-only adenomas, often attributed to larger size and higher prevalence of cavernous sinus invasion [2, 7], whereas others found no significant differences [8, 9]. 

Given these controversies, there is a need for comprehensive evaluation of pathological features in relation to clinical presentation and treatment outcomes. In this retrospective single-center cohort, we systematically assessed IHC staining for GH, PRL, and other anterior pituitary hormones. Our aim was to investigate the associations between these staining patterns, patient characteristics, and treatment responses in order to clarify the prognostic value of pathological subtypes and to inform individualized management strategies in acromegaly.

## Methods

### Study population

Patients diagnosed with acromegaly between 2010 and 2025 were retrospectively reviewed from the database of a tertiary referral hospital. A total of 148 patients who underwent transnasal transsphenoidal surgery (TNTS) with at least 12 months of postoperative follow-up were initially included. Fifty-eight patients were excluded due to the following reasons: prior first-line somatostatin analog (SSA) therapy (*n* = 7), incomplete or absent follow-up data (*n* = 18), unavailability of magnetic resonance imaging (MRI) images with only radiological reports accessible (*n* = 15), incomplete histopathological data (*n* = 8), follow-up duration of less than 12 months (*n* = 4), inability to classify tumor subtype despite re-staining (*n* = 2), and conditions potentially leading to hyperprolactinemia, including interfering medication use, renal failure, or untreated hypothyroidism (*n* = 4).Consequently, 90 patients were eligible for the final analysis. None of the patients received dopamine agonists prior to surgery. Furthermore, no cases of syndromic disease, including AIP- or MEN1-associated adenomas, were identified in the study cohort.

## Diagnostic criteria and biochemical assessment

The diagnosis of acromegaly was established according to international guideline criteria [10]. GH and IGF-1 levels were measured using the Maglumi X3 analyzer (Snibe, Shenzhen, China) with a two-phase enzyme-labeled chemiluminescent immunometric assay. Postoperative biochemical remission was evaluated at 12 weeks after surgery and defined as normalization of age-adjusted serum IGF-1 levels [10]. Where results were borderline, an oral glucose tolerance test (75 g glucose) with GH nadir assessment at 30, 60, 90, and 120 min was additionally performed. Patients without remission were treated based on residual tumor status with surgery, radiosurgery, radiotherapy, or medical therapy. All patients requiring medical therapy received SSAs as first-line agents [1]. Biochemical response to SSA therapy was assessed after at least 3 months of treatment with the maximum recommended doses. Response was defined as normalization of age-adjusted serum IGF-1 levels [10, 11]. Recurrence was defined as elevation of IGF-1 above the age-adjusted upper limit of normal on at least two consecutive measurements following an initial normalization [12]. For analysis, two remission endpoints were defined: early postoperative remission (biochemical remission at 12 weeks after surgery) and long-term remission (biochemical remission at last follow-up after all treatments).

## Histopathological and immunohistochemical analysis

**Fig. 1:**
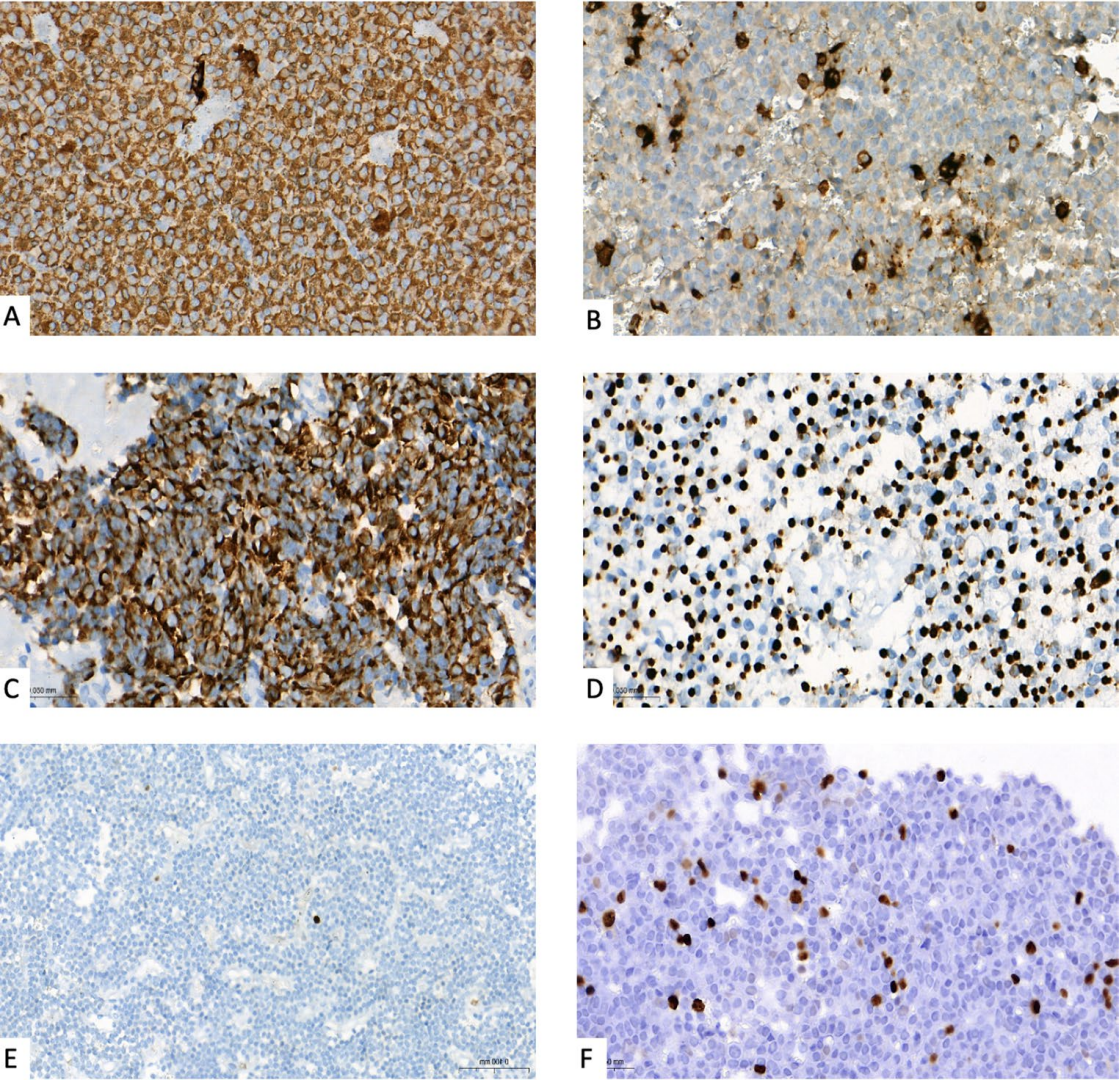
Immunohistochemical features of pituitary adenomas in acromegaly (**A**) Growth hormone (GH) only adenoma showing strong diffuse GH staining. GHx40. (**B**) GH/prolactin (PRL) co-secreting adenoma with scattered moderate and strong cytoplasmic prolactin positive cells. PRLx40. (**C**) Cytokeratin 8/18 (CK8/18) perinuclear pattern consistent with densely granulated subtype. CK8/18 × 40. (**D**) CK8/18 dot-like pattern consistent with sparsely granulated subtype. CK8/18 × 40. (**E**) Ki-67 immunostaining showing low proliferative index (< 3%). Ki67 × 20. (**F**) Ki-67 immunostaining with high proliferative index (≥ 10%). Ki67 × 40

Surgically resected tissues were fixed in 10% buffered formalin, embedded in paraffin, and sectioned at 3–4 μm thickness. Hematoxylin and eosin (H&E) staining was performed, followed by immunohistochemistry (IHC) with indirect biotin-free detection kits (OptiView DAB and UltraView Universal DAB, Ventana, Tucson, USA) on the Ventana Benchmark XT/Ultra platforms (Ventana, Tucson, USA). Antibodies against cytokeratin 8/18 (CK8/18; B22.1&B23.1; Cell Marque), Ki-67 (30 − 9; Ventana-Roche; RRID: AB_2631262), and pituitary hormones (GH [EP267; Cell Marque], PRL[A0569; DAKO; RRID: AB_2893308], TSH[AH21-10; Thermo Fisher Scientific; RRID: AB_62711], FSH [EB257; Cell Marque], LH [Cell Marque; C93], ACTH [Thermo Fisher Scientific; AH26; RRID: AB_61009]) were applied. Hormone positivity was defined as immunoreactivity in ≥ 10% of tumor cells [13]. Tumors were classified as GH-only adenomas (GH only), GH/PRL adenomas (co-expression of GH and PRL), or other (co-expression of GH, PRL, and other pituitary hormones). Primary antibodies against CK8/18 and Ki-67 proliferation labeling index were investigated. CK8/18 immunostaining was evaluated based on staining pattern, and tumors were classified as either perinuclear/diffuse or dot-like (paranuclear, fibrous body type). Accordingly, diffuse perinuclear positivity was defined as densely granulated, whereas a dot-like paranuclear fibrous body pattern was defined as sparsely granulated. The Ki-67 labeling index was determined as the maximum percentage of immunopositive nuclei among at least 1000 counted adenoma cells. Mitotic activity was evaluated by counting mitoses in at least 20 high-power fields at ×400 magnification, corresponding to a total area of 1.8 mm². Antibodies targeting the transcription factors (TFs) PIT1 (POU1F1; Zeca Company), TPIT (TBX19; Gene-Tex), SF1 (EP434; Cell Marque; RRID: AB_2894906), GATA3 (L50-823; Cell Marque; RRID: AB_2895066) were additionally used for histopathological subtyping. Histopathological evaluations and subtyping were done according to recommendations of World Health Organization (WHO) regarding classification of pituitary tumors [14]. All patients harbored PIT-1 lineage PitNETs. Representative immunohistochemical features of pituitary adenomas in patients with acromegaly, including hormone expression patterns, cytokeratin staining subtypes, and Ki-67 proliferation indices, are illustrated in Fig. 1.

## Radiological assessment

All patients underwent contrast-enhanced 3.0-Tesla MRI (Siemens Magnetom Verio, Erlangen, Germany) both pre- and postoperatively. Tumor maximum diameter, optic chiasm compression, and invasion of the cavernous sinus or sphenoid sinus were recorded. Adenomas were classified as microadenomas (< 1 cm) or macroadenomas (≥ 1 cm). The Knosp grading system (grades 0–4) was used to assess cavernous sinus invasion, with grades 3–4 considered invasive [15]. Tumor volume was calculated according to the following formula: height ×width ×length × π/6 [16]. In addition, tumors were qualitatively assessed on T2-weighted sequences and categorized as hyperintense, isointense, or hypointense.

### Statistical analysis

All statistical analyses were conducted using IBM SPSS Statistics version 30 (IBM Corp., Armonk, NY, USA). Histopathological subtype comparisons were performed in 86 patients (GH-only and GH/PRL groups), as the other PIT-1 lineage tumor group (*n* = 4) was excluded from this analysis due to its small sample size. However, those four patients were included in the analyses of postoperative remission outcomes and, where applicable, SSA biochemical response. The normality of continuous variables was assessed both visually and analytically using the Kolmogorov–Smirnov tests. Descriptive statistics were expressed as mean ± standard deviation (SD) for normally distributed variables, median (interquartile range [IQR]) for non-normally distributed variables, and frequency (percentage) for categorical variables. Chi-square or Fisher’s exact tests were used for categorical variables, depending on expected cell counts. For two-group comparisons, Student’s t-test or Mann–Whitney U test was applied as appropriate. Monte Carlo simulation was used when expected counts were low.Both Pearson and Spearman correlation tests were used to evaluate the relationship between continuous variables, depending on the distribution of the data. Univariable and multivariable logistic regression analyses were performed separately to identify factors associated with postoperative remission and SSA response.Variables achieving *p* < 0.05 in univariable analysis were considered candidates for multivariable modeling.Before modeling, multicollinearity was assessed using Spearman correlation, and variables with *r* > 0.6 were not included together.For the postoperative remission model, the final multivariable analysis included optic chiasm compression, T2-MRI hypointensity, cavernous sinus invasion, and preoperative GH level. For the SSA response model, the final multivariable analysis included T2-MRI hypointensity and perinuclear CK8/18 staining pattern. Odds ratios (ORs) and 95% confidence intervals (CIs) were calculated. A two-tailed p-value < 0.05 was considered statistically significant.

## Results

**Fig. 2:**
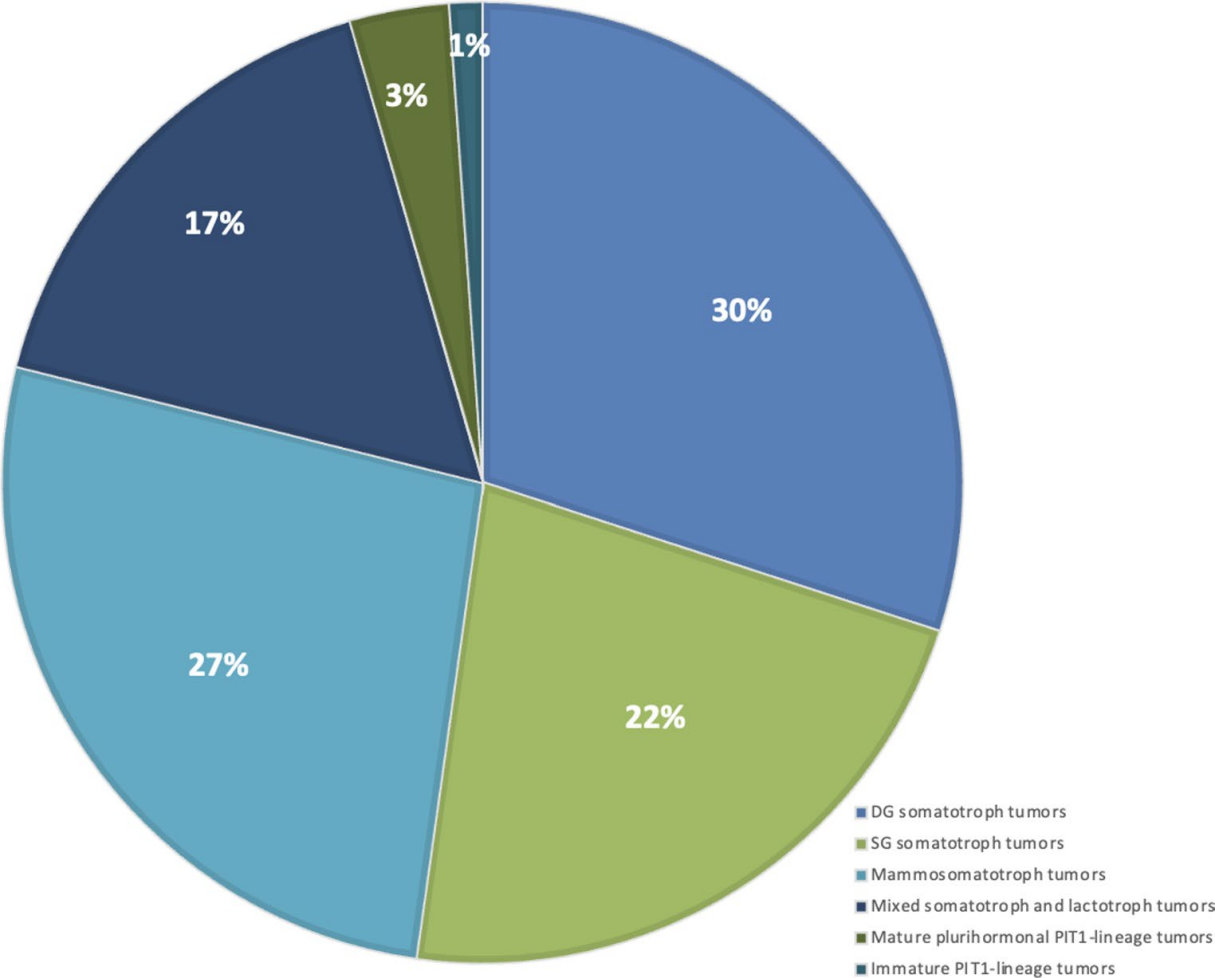
Distribution of hormone staining patterns

The study included 90 (58 females [64.4%] and 32 males [35.6%]) patients with acromegaly. The mean age at diagnosis was 40.69 ± 11.56 years. IHC revealed that 47 patients (52.2%) had GH-only adenomas, comprising 27 densely granulated somatotroph tumors (DGST; 30.0%) and 20 sparsely granulated somatotroph tumors (SGST; 22.2%). Of the remaining 43 patients, 39 (43.3%) had adenomas with co-expression of GH and PRL; comprising 24 (26.7%) mammosomatotroph tumors and 15 (16.7%) mixed somatotroph–lactotroph tumors, and 4 (4.4%) harbored other PIT-1 lineage tumors: three mature plurihormonal PIT-1 lineage tumors (3.3%) and one immature PIT-1 lineage tumor (1.1%) illustrated in Fig. 2.

Baseline characteristics are summarized in Table 1. Preoperative PRL levels were significantly higher in GH/PRL adenomas than in GH-only adenomas (median 32.00 vs. 15.20 ng/mL; *p* < 0.001), and the prevalence of PRL > 100 ng/mL was also significantly higher in the GH/PRL group (25.6% vs. 4.3%; *p* = 0.010). In histopathological evaluation, the Ki-67 proliferation index was significantly higher in GH/PRL adenomas than in GH-only adenomas (4.31 ± 2.33% vs. 3.45 ± 3.01%; *p* = 0.009). Postoperatively, SSA therapy was administered to 21 patients (44.7%) in the GH-only group and 22 (56.4%) in the GH/PRL group (*p* = 0.386), while cabergoline was used in 2 (4.3%) and 7 (17.9%) patients, respectively (*p* = 0.073).Within the GH-only group, SGST were diagnosed at a significantly younger age than DGST (35.90 ± 10.02 vs. 43.30 ± 11.43 years; *p* = 0.018) and were characterized by larger tumor size (24.20 ± 13.90 vs. 14.70 ± 6.92 mm; *p* = 0.005), greater tumor volume (median 2.26 vs. 0.65 cm³; *p* = 0.007), and a higher rate of macroadenomas (100% vs. 81.5%; *p* = 0.031). SGST also showed higher rates of cavernous sinus invasion (40.0% vs. 7.4%; *p* = 0.011) and optic chiasm compression (50.0% vs. 22.2%; *p* = 0.047), and a less favorable Knosp score distribution (*p* = 0.037). On MRI, T2 hypointensity was more frequent in DGST than in SGST (51.9% vs. 15.0%; *p* = 0.014). Histopathologically, SGST exhibited a significantly higher Ki-67 index (5.25 ± 3.78% vs. 2.19 ± 1.08%; *p* = 0.001) and a higher rate of Ki-67 ≥ 10% (20.0% vs. 0%; *p* = 0.027). Postoperatively, SGST had higher residual tumor rates (80.0% vs. 40.7%; *p* = 0.009), lower early remission rates (15.0% vs. 51.9%; *p* = 0.006), and higher postoperative IGF-1 levels (median 575.00 vs. 318.50 ng/mL; *p* = 0.003). At final follow-up, GH and IGF-1 levels remained significantly higher in SGST compared with DGST (median 0.80 vs. 0.33 ng/mL, *p* = 0.023; and 187.00 vs. 132.00 ng/mL, *p* = 0.034, respectively). No significant differences were identified between mammosomatotroph and mixed somatotroph–lactotroph adenomas in any of the evaluated parameters.

**Table 1:** Baseline characteristics, imaging, histopathology, treatment modalities, and outcomes of patients with GH-only and GH/PRL adenomas

Variable	GH-only (*n* = 47)				GH/PRL (*n* = 39)				*p* value
		Densely granulated somatotrophs (*n* = 27)	Sparsely granulated somatotrophs (*n* = 20)	*p* value		Mammosomatotrophs (*n* = 24)	Mixed somatotroph – lactotrophs (*n* = 15)	*p* value	
Age, years (mean ± SD)	39.72 ± 10.96	43.30 ± 11.43	35.90 ± 10.02	**0.018**	40.67 ± 11.99	42.58 ± 10.97	37.60 ± 13.28	0.184	0.667
Gender, female, n (%)	34 (72.3%)	21 (77.8%)	13 (65.0%)	0.333	22 (56.4%)	11 (45.8%)	11 (73.3%)	0.092	0.123
BMI, kg/m^2^ (mean ± SD)	27.82 ± 3.76	28.41 ± 3.36	26.88 ± 4.17	0.337	28.79 ± 4.03	29.52 ± 4.06	27.60 ± 3.83	0.168	0.289
Preoperation									
GH, ng/mL, (median [IQR])	9.00 [4.34–22.50]	7.00 [3.55–9.50]	11.20 [7.00–24.70]	0.112	7.00 [3.80–16.85]	8.0 [2.5–23.0]	6.1 [3.6–8.3]	0.199	0.904
IGF-1, ng/mL (median [IQR])	790 [523–1300]	786.00 [521.00–944.50]	982.00 [529.75–1575.00]	0.146	760 [487.5–1332.5]	729 [423–1365]	817 [564–1255]	0.684	0.904
IGF-1, %ULN, (median [IQR])	2.40 [1.90–3.90]	2.40 [2.00–3.65]	2.85 [1.43–4.00]	0.796	2.50 [2.25–5.10]	2.50 [1.50–5.10]	2.45 [1.35–4.15]	0.863	0.904
Nadir GH, ng/mL, (median [IQR])	13.10 [4.10–17.70]	16.90 [7.00–24.95]	7.10 [4.10–11.10]	0.245	3.80 [2.25–15.05]	4.45 [1.53–15.05]	3.00 [2.25–14.40]	0.734	0.139
PRL, ng/mL, (median [IQR])	15.20 [10.40–23.00]	14.00 [7.10–21.00]	17.20 [14.10–31.90]	0.106	32.00 [18.00–100.00]	49.50 [17.25–103.75]	20.00 [19.00–78.00]	0.363	**< 0.001**
PRL > 200 ng/mL, n (%)	1 (2.1%)	1 (3.7%)	0 (0.0%)	1.000	2 (5.1%)	1 (4.2%)	1 (6.7%)	0.731	0.588
PRL > 100 ng/mL, n (%)	2 (4.3%)	2 (7.4%)	0 (0.0%)	0.500	10 (25.6%)	7 (29.2%)	3 (20.0%)	0.524	**0.010**
TSH deficit, n (%)	7 (14.9%)	4 (14.8%)	3 (15.0%)	1.000	5 (12.8%)	3 (12.5%)	2 (13.3%)	0.940	1.000
ACTH deficit, n (%)	6 (12.8%)	2 (7.4%)	4 (20.0%)	0.379	3 (7.7%)	3 (12.5%)	0 (0.0%)	0.271	0.502
FSH/LH deficit, n (%)	14 (29.8%)	9 (33.3%)	5 (25.0%)	0.748	14 (35.9%)	10 (41.7%)	4 (26.7%)	0.342	0.646
Hypopituitarism, n (%)	20 (42.6%)	11 (40.7%)	9 (45.0%)	0.652	16 (41.0%)	11 (45.8%)	5 (33.3%)	0.440	1.000
Imaging									
Maximum tumor size, mm, (mean ± SD)	19.00 ± 11.22	14.70 ± 6.92	24.20 ± 13.90	**0.005**	19.82 ± 11.78	22.25 ± 13.33	15.93 ± 7.64	0.219	0.941
Tumor volume, cm^3^, (median [IQR])	0.84 [0.50–3.60]	0.65 [0.29–1.35]	2.26 [0.67–7.05]	**0.007**	1.25 [0.36–6.00]	1.49 [0.44–6.45]	1.00 [0.14–4.80]	0.341	0.716
Macroadenoma, n (%)	42 (89.4%)	22 (81.5%)	20 (100.0%)	**0.031**	31 (79.5%)	20 (83.3%)	11 (73.3%)	0.452	0.238
Knosp score, n (%)				**0.037**				0.131	
0	15 (31.9%)	11 (40.7%)	4 (20.0%)		14 (35.9%)	7 (29.2%)	7 (46.7%)		0.559
1	13 (27.7%)	10 (37.0%)	3 (15.0%)		7 (17.9%)	5 (20.8%)	2 (13.3%)		
2	9 (19.1%)	4 (14.8%)	5 (25.0%)		8 (20.5%)	5 (20.8%)	3 (20.0%)		
3	7 (14.9%)	1 (3.7%)	6 (30.0%)		4 (10.3%)	1 (4.2%)	3 (20.0%)		
4	3 (6.4%)	1 (3.7%)	2 (10.0%)		6 (15.4%)	6 (25.0%)	0 (0.0%)		
Optic chiasm compression, n (%)	16 (34.0%)	6 (22.2%)	10 (50.0%)	**0.047**	14 (35.9%)	10 (41.7%)	4 (26.7%)	0.342	1.000
Cavernous sinus invasion, n (%)	10 (21.3%)	2 (7.4%)	8 (40.0%)	**0.011**	10 (25.6%)	7 (29.2%)	3 (20.0%)	0.524	0.798
Sphenoid sinus invasion, n (%)	10 (21.3%)	5 (18.5%)	5 (25.0%)	0.723	12 (30.8%)	9 (37.5%)	3 (20.0%)	0.249	0.333
T2-MRI hypointensity, n (%)	17 (36.2%)	14 (51.9%)	3 (15.0%)	**0.014**	21 (53.8%)	14 (58.3%)	7 (46.7%)	0.477	0.128
Histopathology									
Mitotic index, (median [IQR])	0 [0–1]	0 [0–0]	0 [0–1]	0.333	0 [0–0]	0 [0–0]	0 [0–1]	0.046	0.177
Ki 67 index, %, (mean ± SD)	3.45 ± 3.01	2.19 ± 1.08	5.25 ± 3.78	**0.001**	4.31 ± 2.33	4.46 ± 2.28	4.07 ± 2.46	0.419	**0.009**
Ki 67 ≥ 3%, (n, %)	21 (44.7%)	7 (25.9%)	14 (70.0%)	**0.003**	30 (76.9%)	21 (87.5%)	9 (60.0%)	0.063	**0.003**
Ki 67 ≥ 10%, (n, %)	4 (8.5%)	0 (0.0%)	4 (20.0%)	**0.027**	1 (2.6%)	1 (4.2%)	0 (0.0%)	0.423	0.371
Perinuclear CK8/18 staining, (n, %)	27 (57.4%)	27 (100.0%)	0 (0.0%)	**< 0.001**	15 (38.5%)	10 (41.7%)	5 (33.3%)	0.603	0.088
Postoperation									
GH, ng/mL, (median [IQR])	2.10 [0.95–4.55]	1.15 [0.55–4.25]	2.50 [1.54–5.00]	0.103	1.10 [0.80–2.15]	1.30 [0.90–2.30]	1.10 [0.70–2.13]	0.684	0.129
IGF-1, ng/mL, (median [IQR])	365 [212–590.5]	318.50 [139.25–421.00]	575.00 [379.00–1158.50]	**0.003**	306 [183–500.5]	260 [156–583]	351 [196–483]	0.778	0.324
IGF-1, %ULN, (median [IQR])	1.40 [0.90–2.25]	1.05 [0.65–1.60]	2.00 [1.15–4.35]	**0.014**	1.10 [0.85–1.95]	1.10 [0.90–2.00]	1.20 [0.80–1.93]	0.875	0.483
Residual tumor, (n, %)	27 (57.4%)	11 (40.7%)	16 (80.0%)	**0.009**	24 (61.5%)	15 (62.5%)	9 (60.0%)	0.876	0.826
Maximum residual tumor size, mm, (mean ± SD)	14.71 ± 11.42	12.25 ± 6.92	16.23 ± 13.53	0.689	11.35 ± 8.20	12.92 ± 9.77	9.00 ± 4.69	0.588	0.187
Remission, (n, %)	17 (36.2%)	14 (51.9%)	3 (15.0%)	**0.006**	15 (38.5%)	9 (37.5%)	6 (40.0%)	0.876	1.000
TSH deficit, n (%)	10 (21.3%)	6 (22.2%)	4 (20.0%)	1.000	8 (20.5%)	5 (20.8%)	3 (20.0%)	0.950	1.000
ACTH deficit, n (%)	9 (19.1%)	5 (18.5%)	4 (20.0%)	1.000	6 (15.4%)	4 (16.7%)	2 (13.3%)	0.779	0.778
FSH/LH deficit, n (%)	12 (25.5%)	6 (22.2%)	6 (30.0%)	0.737	11 (28.2%)	8 (33.3%)	3 (20.0%)	0.368	0.811
AVP deficit, n (%)	5 (10.6%)	2 (7.4%)	3 (15.0%)	0.638	7 (17.9%)	6 (25.0%)	1 (6.7%)	0.147	0.365
Hypopituitarism, n (%)	18 (38.3%)	11 (40.7%)	7 (35.0%)	0.688	13 (33.3%)	9 (37.5%)	4 (26.7%)	0.485	0.659
Recurrence, n (%)	1 (2.1%)	1 (3.7%)	0 (0.0%)	1.000	2 (5.1%)	2 (8.3%)	0 (0.0%)	0.251	0.588
Second-line treatment									
SSA, n (%)	21 (44.7%)	11 (40.7%)	10 (50.0%)	0.566	22 (56.4%)	13 (54.2%)	9 (60.0%)	0.721	0.386
Cabergoline, n (%)	2 (4.3%)	0 (0.0%)	2 (10.0%)	0.176	7 (17.9%)	4 (16.7%)	3 (20.0%)	0.792	0.073
Reoperation, n (%)	6 (12.8%)	2 (7.4%)	4 (20.0%)	0.379	2 (5.1%)	2 (8.3%)	0 (0.0%)	0.251	0.283
Gamma knife, n (%)	2 (4.3%)	0 (0.0%)	2 (10.0%)	0.176	0 (0.0%)	0 (0.0%)	0 (0.0%)	—	0.498
Conventional RT, n (%)	1 (2.1%)	0 (0.0%)	1 (5.0%)	0.426	0 (0.0%)	0 (0.0%)	0 (0.0%)	—	1.000
Follow - up									
Remission at last follow up, n (%)	41 (87.2%)	26 (96.3%)	15 (75.0%)	0.070	37 (94.9%)	23 (95.8%)	14 (93.3%)	0.731	0.283
GH, ng/mL, (median [IQR])	0.50 [0.30–1.20]	0.33 [0.20–0.88]	0.80 [0.42–1.95]	**0.023**	0.60 [0.30–1.21]	0.60 [0.26–1.40]	0.60 [0.30–0.80]	0.718	0.655
IGF-1, ng/mL, (median [IQR])	141 [93.8–204]	132.00 [88.00–175.00]	187.00 [103.25–315.00]	**0.034**	154 [110–220]	144 [104–213]	163 [111–228]	0.341	0.594
IGF-1, %ULN, (median [IQR])	0.60 [0.40–0.80]	0.50 [0.40–0.80]	0.70 [0.50–1.05]	0.106	0.70 [0.40–0.80]	0.60 [0.43–0.80]	0.70 [0.30–0.80]	0.805	0.678
Follow up duration, years (mean ± SD)	10.14 ± 4.25	10.76 ± 4.14	9.23 ± 4.30	0.282	9.33 ± 4.40	9.27 ± 4.00	9.42 ± 5.12	0.840	0.284

*ACTH* adrenocorticotropic hormone, *AVP* arginine vasopressin, *BMI* body mass index, *CK8/18* cytokeratin 8/18, *FSH* follicle-stimulating hormone, *GH* growth hormone, *IGF-1* insulin-like growth factor-1, *PRL* prolactin, *RT* radiotherapy, *SSA* somatostatin analog, *TSH* thyroid-stimulating hormone

Postoperative remission was achieved in 32 patients (35.6%). Remission rates were 36.2% in GH-only, 38.5% in GH/PRL, and 0% in other PIT-1 lineage tumors (*p* = 0.308). DGST were significantly more frequent in patients with postoperative remission compared with those without remission (83.3% vs. 41.4%, *p* = 0.006), whereas the proportion of mammosomatotroph tumors was similar between the two groups (60.0% vs. 62.5%, *p* = 0.876). Compared with those without remission, patients in remission had lower preoperative GH (7.0 vs. 12.0 ng/mL, *p* = 0.026) and PRL levels (15.6 vs. 28.0 ng/mL, *p* = 0.004), and smaller adenomas (13.34 vs. 22.26 mm, *p* < 0.001). Optic chiasm compression (9.4% vs. 50.0%, *p* < 0.001) and cavernous sinus invasion (6.3% vs. 31.0%, *p* = 0.007) was more frequent in non-remission cases, whereas T2 hypointensity was more common in remission patients (65.6% vs. 34.5%, *p* = 0.008). Two patients (6.3%) in the remission group and one (1.7%) in the non-remission group experienced recurrence after surgery (*p* = 0.287). (Table 2). In univariable logistic regression, higher preoperative GH level (OR 0.935, 95% CI 0.885–0.988, *p* = 0.016), macroadenoma (OR 0.189, 95% CI 0.053–0.677, *p* = 0.011), cavernous sinus invasion (OR 0.148, 95% CI 0.032–0.688, *p* = 0.015), and optic chiasm compression (OR 0.103, 95% CI 0.028–0.378, *p* < 0.001) were all associated with reduced odds of remission, while T2-MRI hypointensity was positively associated with remission (OR 3.627, 95% CI 1.462–8.998, *p* = 0.005). In the multivariable model, optic chiasm compression was independently associated with postoperative remission (OR 0.212, 95% CI 0.049–0.910, *p* = 0.037), whereas T2-MRI hypointensity, cavernous sinus invasion, and preoperative GH level did not reach statistical significance.

**Table 2:** Comparison of baseline, imaging, histopathological, and postoperative characteristics between patients with and without early postoperative remission

Variable	Post-op remission (*n* = 32)	Post-op non-remission (*n* = 58)	*P* value
Age, years (mean ± SD)	43.78 ± 10.88	38.98 ± 11.67	0.052
Gender, female, n (%)	20 (62.5%)	38 (65.5%)	0.775
BMI, kg/m^2^ (mean ± SD)	28.34 ± 4.18	28.41 ± 3.84	0.927
Preoperation			
GH, ng/mL, (median [IQR])	7.0 [3.88–8.0]	12.0 [4.23–24.55]	**0.026**
IGF-1, ng/mL (median [IQR])	712 [518.3–910.3]	892.5 [545–1353.8]	0.096
IGF-1, %ULN, (median [IQR])	2.20 [1.68–3.63]	2.85 [1.60–4.30]	0.291
Nadir GH, ng/mL, (median [IQR])	4.15 [1.70–13.05]	4.70 [2.50–17.00]	0.626
PRL, ng/mL, (median [IQR])	15.6 [10.80–20]	28 [15.23–69]	**0.004**
PRL > 200 ng/mL, n (%)	1 (3.1%)	2 (3.4%)	1.000
PRL > 100 ng/mL, n (%)	2 (6.3%)	10 (17.2%)	0.201
TSH deficit, n (%)	3 (9.4%)	10 (17.2%)	0.366
ACTH deficit, n (%)	1 (3.1%)	8 (13.8%)	0.150
FSH/LH deficit, n (%)	10 (31.3%)	19 (32.8%)	1.000
Hypopituitarism, n (%)	12 (37.5%)	26 (44.8%)	0.656
Imaging			
Maximum tumor size, mm, (mean ± SD)	13.34 ± 7.54	22.26 ± 11.75	**< 0.001**
Tumor volume, cm^3^, (median [IQR])	0.51 [0.11–1.00]	1.74 [0.65–6.68]	**< 0.001**
Macroadenoma, n (%)	23 (71.9%)	54 (93.1%)	**0.011**
Knosp score, n (%)			**< 0.001**
0	20 (62.5%)	9 (15.5%)	
1	7 (21.9%)	15 (25.9%)	
2	3 (9.4%)	16 (27.6%)	
3	2 (6.3%)	9 (15.5%)	
4	0 (0.0%)	9 (15.5%)	
Optic chiasm compression, n (%)	3 (9.4%)	29 (50.0%)	**< 0.001**
Cavernous sinus invasion, n (%)	2 (6.3%)	18 (31.0%)	**0.007**
Sphenoid sinus invasion, n (%)	4 (12.5%)	18 (31.0%)	0.072
T2-MRI hypointensity, n (%)	21 (65.6%)	20 (34.5%)	**0.008**
Histopathology			
Mitotic index, (median [IQR])	0 [0–1]	0 [0–1]	0.851
Ki 67 index, %, (mean ± SD)	3.03 ± 1.93	4.19 ± 3.02	0.087
Ki 67 ≥ 10%, (n, %)	0 (0.0%)	5 (8.6%)	0.156
Perinuclear CK8/18 staining, (n, %)	20 (62.5%)	25 (43.1%)	0.123
Dens granulated somatotrophs, n (%) *	14 (82.3%)	13 (43.3%)	**0.012**
Mammosomatotrophs, n (%) **	9 (60.0%)	15 (62.5%)	0.876
Postoperation			
GH, ng/mL, (median [IQR])	0.90 [0.25–1.25]	2.30 [1.15–4.95]	**< 0.001**
IGF-1, ng/mL, (median [IQR])	151 [116–232]	470 [369.5–665.5]	**< 0.001**
IGF-1, %ULN, (median [IQR])	0.80 [0.55–1.00]	1.90 [1.40–2.75]	**< 0.001**
Residual tumor, (n, %)	2 (6.3%)	51 (87.9%)	**< 0.001**
Maximum residual tumor size, mm, (mean ± SD)	—	13.02 ± 9.89	—
TSH deficit, n (%)	5 (15.6%)	13 (22.4%)	0.585
ACTH deficit, n (%)	3 (9.4%)	1 (1.7%)	0.240
FSH/LH deficit, n (%)	5 (15.6%)	20 (34.5%)	0.084
AVP deficit, n (%)	2 (6.3%)	10 (17.2%)	0.201
Hypopituitarism, n (%)	7 (21.9%)	26 (44.8%)	**0.040**
Recurrence, n (%)	2 (6.3%)	1 (1.7%)	0.287
Follow - up			
Remission at last follow up, n (%)	32 (100.0%)	47 (81.0%)	**0.007**
GH, ng/mL, (median [IQR])	0.30 [0.11–0.73]	0.80 [0.38–1.63]	**< 0.001**
IGF-1, ng/mL, (median [IQR])	133.5 [90.9–162.3]	174 [108.3–240.3]	**0.006**
IGF-1, %ULN, (median [IQR])	0.50 [0.40–0.70]	0.70 [0.50–1.00]	**0.008**
Follow up duration, years (mean ± SD)	9.75 ± 4.66	9.60 ± 4.15	0.909

*ACTH* adrenocorticotropic hormone, *AVP* arginine vasopressin, *BMI* body mass index, *CK8/18* cytokeratin 8/18, *FSH* follicle-stimulating hormone, *GH* growth hormone, *IGF-1* insulin-like growth factor-1, *PRL* prolactin, *RT* radiotherapy, *TSH* thyroid-stimulating hormone

*Analyses were performed for n = 47

**Analyses were performed for n = 39

Among 45 patients who received SSA therapy, 25 (55.6%) achieved a biochemical response. SSA response rates were 42.9% in GH-only, 68.2% in GH/PRL, and 50.0% in other PIT-1 lineage tumors (*p* = 0.245). DGST were more frequent in responders (87.5% vs. 20.0%, *p* = 0.020), while mammosomatotrophs also tended to be more common (73.3% vs. 28.6%, *p* = 0.074). Responders were older (40.0 vs. 34.5 years, *p* = 0.027), had lower baseline IGF-1 (591 vs. 1055 ng/mL, *p* = 0.040), and smaller adenomas (18.1 vs. 24.0 mm, *p* = 0.010). They also showed a higher frequency of T2 hypointensity (56.0% vs. 20.0%, *p* = 0.018) and perinuclear CK8/18 staining (60.0% vs. 25.0%, *p* = 0.034), and a lower Ki-67 index (3.52% vs. 5.80%, *p* = 0.041). Postoperative IGF-1 levels were significantly lower in responders (381 vs. 553 ng/mL, *p* = 0.004), as was maximum residual tumor size (8.1 vs. 13.8 mm, *p* = 0.005). At last follow-up, remission was more frequent in responders (100% vs. 70.0%, *p* = 0.005), and IGF-1 levels remained significantly lower (155 vs. 191 ng/mL, *p* = 0.049) (Table 3).In univariable logistic regression, older age (OR 1.072, 95% CI 1.006–1.143, *p* = 0.033), T2-MRI hypointensity (OR 5.091, 95% CI 1.319–19.649, *p* = 0.018), and perinuclear CK8/18 staining (OR 4.500, 95% CI 1.238–16.351, *p* = 0.022) were significantly associated with SSA response. In the multivariable model, both perinuclear CK8/18 staining (OR 4.839, 95% CI 1.198–19.540, *p* = 0.027) and T2-MRI hypointensity (OR 5.464, 95% CI 1.284–23.250, *p* = 0.022) were independently associated with SSA response.

**Table 3:** Comparison of baseline, imaging, histopathological, and postoperative characteristics between patients with and without SSA response

Variable	SSA responders (*n* = 25)	SSA non-responders (*n* = 20)	
Age, years (mean ± SD)	40.0 ± 8.9	34.5 ± 12.3	**0.027**
Gender, female, n (%)	16 (64.0%)	15 (75.0%)	0.428
BMI, kg/m^2^ (mean ± SD)	29.2 ± 4.2	27.4 ± 3.8	0.220
Preoperation			
GH, ng/mL, (median [IQR])	7.4 [2.0–19.9]	11.2 [4.5–26.0]	0.272
IGF-1, ng/mL (median [IQR])	591 [447–1313]	1055 [790–1800]	**0.040**
IGF-1, %ULN, (median [IQR])	2.5 [1.5–4.5]	3.0 [2.1–4.0]	0.628
Nadir GH, ng/mL, (median [IQR])	2.7 [2.1–4.5]	16.9 [2.7–18.1]	0.142
PRL, ng/mL, (median [IQR])	28.0 [18.5–101.5]	25.4 [15.0–48.3]	0.326
PRL > 200 ng/mL, n (%)	2 (8.0%)	0 (0.0%)	0.495
PRL > 100 ng/mL, n (%)	7 (28.0%)	1 (5.0%)	0.059
TSH deficit, n (%)	3 (12.0%)	6 (30.0%)	0.157
ACTH deficit, n (%)	3 (12.0%)	4 (20.0%)	0.682
FSH/LH deficit, n (%)	6 (24.0%)	10 (50.0%)	0.117
Hypopituitarism, n (%)	10 (40.0%)	12 (60.0%)	0.236
Imaging			
Maximum tumor size, mm, (mean ± SD)	18.1 ± 10.8	24.0 ± 9.2	**0.010**
Tumor volume, cm^3^, (median [IQR])	0.71 [0.43–3.43]	3.58 [1.28–9.30]	**0.033**
Macroadenoma, n (%)	21 (84.0%)	20 (100.0%)	0.117
Knosp score, n (%)			0.061
0	8 (32.0%)	2 (10.0%)	
1	8 (32.0%)	3 (15.0%)	
2	5 (20.0%)	8 (40.0%)	
3	1 (4.0%)	5 (25.0%)	
4	3 (12.0%)	2 (10.0%)	
Optic chiasm compression, n (%)	10 (40.0%)	10 (50.0%)	0.557
Cavernous sinus invasion, n (%)	4 (16.0%)	7 (35.0%)	0.176
Sphenoid sinus invasion, n (%)	6 (24.0%)	6 (30.0%)	0.741
T2-MRI hypointensity, n (%)	14 (56.0%)	4 (20.0%)	**0.018**
Histopathology			
Mitotic index, (median [IQR])	0 [0–1]	0 [0–1]	0.304
Ki 67 index, %, (mean ± SD)	3.52 ± 2.14	5.80 ± 3.76	**0.041**
Ki 67 ≥ 10%, (n, %)	0 (0.0%)	4 (20.0%)	**0.033**
Perinuclear CK8/18 staining, n (%)	15 (60%)	5 (25.0%)	**0.034**
Dens granulated somatotrophs, n (%) *	7 (87.5%)	3 (23.1%)	**0.020**
Mammosomatotrophs, n (%) **	11 (73.3%)	2 (28.6%)	0.074
Postoperation			
GH, ng/mL, (median [IQR])	1.7 [1.0–2.5]	2.5 [1.4–6.0]	0.059
IGF-1, ng/mL, (median [IQR])	381 [312–436]	553 [424–680]	**0.004**
IGF-1, %ULN, (median [IQR])	1.6 [1.1–1.9]	2.0 [1.3–2.9]	0.138
Residual tumor, (n, %)	18 (72.0%)	7 (35.0%)	0.059
Maximum residual tumor size, mm, (mean ± SD)	8.1 ± 7.2	13.8 ± 9.0	**0.005**
TSH deficit, n (%)	6 (24.0%)	5 (25.0%)	1.000
ACTH deficit, n (%)	4 (16.0%)	4 (20.0%)	1.000
FSH/LH deficit, n (%)	6 (24.0%)	8 (40.0%)	0.336
AVP deficit, n (%)	5 (20.0%)	3 (15.0%)	0.716
Hypopituitarism, n (%)	9 (36.0%)	10 (50.0%)	0.379
Follow - up			
Remission at last follow up, n(%)	25 (100.0%)	14 (70.0%)	**0.005**
GH, ng/mL, (median [IQR])	0.70 [0.30–1.35]	1.15 [0.56–3.05]	0.093
IGF-1, ng/mL, (median [IQR])	155 [92–188]	191 [111–424]	**0.049**
IGF-1, %ULN, (median [IQR])	0.70 [0.40–0.85]	0.70 [0.33–1.10]	0.687
Follow up duration, years (mean ± SD)	11.02 ± 4.00	8.25 ± 3.31	**0.035**

*ACTH* adrenocorticotropic hormone, *AVP* arginine vasopressin, *BMI* body mass index, *CK8/18* cytokeratin 8/18, *FSH* follicle-stimulating hormone, *GH* growth hormone, *IGF-1* insulin-like growth factor-1, *PRL* prolactin, *RT* radiotherapy, *TSH* thyroid-stimulating hormone

*Analyses were performed for n = 21

**Analyses were performed for n = 22

In correlation analyses, tumor volume did not show significant correlations with baseline GH, PRL and IGF-1 in overall cohort. (rho = 0.201, *p* = 0.065; rho = 0.126, *p* = 0.132, rho = 0.105, *p* = 0.343, respectively). Similarly, no significant correlations were observed between tumor volume and GH (rho = 0.112, *p* = 0.480), IGF-1 (rho = − 0.056, *p* = 0.719), or PRL (rho = 0.158, *p* = 0.290) in the GH-only tumor subgroup. In contrast, within the GH/PRL tumor subgroup, tumor volume correlated positively with both baseline PRL (rho = 0.395, *p* = 0.013) and GH (rho = 0.348, *p* = 0.035), whereas no significant association was found with IGF-1 (rho = 0.268, *p* = 0.108).

## Discussion

In our study, GH-only adenomas accounted for 52.2% of cases, GH/PRL adenomas for 43.3%, and other PIT-1 lineage tumors for 4.4%. Patients with GH/PRL adenomas exhibited significantly higher Ki-67 indices, indicating greater proliferative activity, but surgical remission rates were comparable between GH/PRL and GH-only groups. Interestingly, GH/PRL adenomas showed a tendency toward higher SSA responsiveness.

In the most recent WHO classification, pituitary adenomas are categorized according to their cell lineage (PIT-1, TPIT, and SF1) [17]. While transcription factor staining provides important information for broad tumor classification, PIT-1 immunopositivity alone does not distinguish between GH-only and GH/PRL adenomas, as both share this lineage marker. Although not yet completely established, estrogen receptor alpha (ERα) is included in the WHO classification as a transcription factor to further differentiate pure GH from GH/PRL adenomas. However, ERα staining was not available in our series, and therefore tumor subclassification was based on hormonal immunoreactivity alone. While the pathological definition of GH/PRL adenomas is established within the most recent WHO classification, there is currently no universally accepted clinical and biochemical criteria of GH/PRL adenomas, and reported prevalence varies widely depending on the criteria used [18]. Our classification was based exclusively on PRL immunoreactivity, independent of serum PRL levels. In our cohort, the GH/PRL group represented 43.3% of cases consistent with other IHC-based series reporting 24–43.6% [2, 3, 19, 20], but exceeding estimates from studies requiring concomitant serum PRL elevation [7, 8, 21] This lack of consensus is mainly due to the difficulty in distinguishing true cosecretion from hyperprolactinemia secondary to stalk effect. Some authors have suggested a PRL cut-off > 100 ng/mL as indicative of adenoma secretion [4], whereas the Pituitary Society consensus emphasizes that PRL < 200 ng/mL is less likely to reflect true secretion [22]. Both thresholds remain imperfect: tumors with stalk effect may exceed 100 ng/mL, and some genuine cosecreting adenomas may present with normal PRL levels. In our series, median PRL levels were significantly higher in GH/PRL adenomas compared with GH-only adenomas, however when applying cut-offs, 13.3% of patients had PRL > 100 ng/mL; significantly more frequent in GH/PRL group than in GH-only group (25.6% vs. 4.3%, *p* = 0.010) whereas only 3.3% had PRL > 200 ng/mL, with no significant group differences.

In our cohort, the mean age at diagnosis was 40 years, and females accounted for approximately 64.4% of cases. No significant age or sex differences were observed between GH-only and GH/PRL adenomas. Some earlier reports suggested that GH/PRL adenomas tend to occur in younger patients, particularly in those with childhood-onset gigantism [9], and that acromegaly with hyperprolactinemia may present 3–5 years earlier than GH-only adenomas [7, 8]. A recent multicenter study from Spain reported a younger mean age in GH/PRL compared with GH-only adenomas (43.5 vs. 50.1 years, *p* = 0.006) [21] Regarding gender, a large cohort of 529 patients found a higher proportion of females in acromegaly with hyperprolactinemia compared with those with normal PRL (64.7% vs. 50%, *p* = 0.001) [7]. However, IHC-based series failed to confirm such differences, reporting similar age and gender distribution across GH-only and GH/PRL adenomas [2, 3, 19, 23].

Regarding hormonal parameters, baseline GH, nadir GH, IGF-1, and IGF-1 index values did not differ significantly between groups in our cohort, and the prevalence of hypopituitarism remained comparable. Previous studies have reported inconsistent findings in patients with GH/PRL adenomas. Some series described significantly higher serum IGF-1 and GH levels, including higher nadir GH values during OGTT, in dual-staining adenomas [3, 17, 21], whereas others did not detect baseline differences across histological subtypes [19, 23]. In contrast, higher baseline GH levels were observed in some cohorts of patients without hyperprolactinemia [8].

No differences in tumor diameter or prevalence of invasive disease were observed between GH-only and GH/PRL adenomas in our series. These results contrast with some serum-based series suggesting larger and more invasive GH/PRL adenomas [7, 8], though IHC-based studies have reported more variable findings [2, 3, 19, 20]. A likely explanation for these conflicting findings lies in the varying proportions of mammosomatotroph tumors included across studies. Lv et al. showed that mixed somatotroph-lactotroph adenomas are aggressive tumors, typically associated with larger size, greater invasiveness, and lower biochemical remission rates [2]. 

In our cohort, GH/PRL adenomas exhibited a significantly higher mean Ki-67 index compared with GH-only adenomas (4.31% vs. 3.45%, *p* = 0.009), and the rate of Ki-67 ≥ 3% was also significantly more frequent in the GH/PRL group (76.9% vs. 44.7%, *p* = 0.003), though the rate of Ki-67 ≥ 10% did not differ significantly between groups (2.6% vs. 8.5%, *p* = 0.371). Importantly, Ki-67 status was not significantly associated with postoperative remission or SSA responsiveness in univariate or multivariate analyses. Instead, classical clinical and radiological factors determined outcomes. For postoperative remission, optic chiasm compression emerged as the only independently associated factor (OR 0.212, 95% CI 0.049–0.910, *p* = 0.037). Likewise, SSA response was primarily influenced by perinuclear CK8/18 staining (OR 4.839, 95% CI 1.198–19.540, *p* = 0.027) and T2-MRI hypointensity (OR 5.464, 95% CI 1.284–23.250, *p* = 0.022). These findings indicate that Ki-67, although elevated in GH/PRL adenomas, does not independently predict surgical or medical outcomes. Rather, remission and treatment response in acromegaly are more strongly determined by anatomical invasion, postoperative hormonal levels, and cytokeratin staining patterns.

Whether PRL co-secretion in acromegaly influences surgical and medical outcomes remains debated. In our cohort of 90 patients, postoperative remission was achieved in 35.6% of cases overall, with SSA therapy administered to 50% of patients, among whom 55.6% achieved biochemical response. This postoperative remission rate likely reflects the challenging case mix of our tertiary referral center, predominantly composed of macroadenomas (84.4%) with a high prevalence of cavernous sinus invasion and optic chiasm compression, and is consistent with contemporary large surgical series [24, 25]. Meta-analyses estimate an overall pooled surgical remission rate of approximately 55%, decreasing to 29–37% in invasive macroadenomas [26]. Initial postoperative remission rates were nearly identical between GH-only and GH/PRL groups. Although the overall use of SSAs and adjunctive cabergoline was numerically higher in GH/PRL adenomas compared with GH-only adenomas (56.4% vs. 44.7% and 17.9% vs. 4.3%, respectively), these differences did not reach statistical significance. Notably, SSA response rates showed a trend toward being higher in patients with GH/PRL adenomas compared with GH-only tumors (68.2% vs. 42.9%). However, this association did not fully retain significance in logistic regression analyses, suggesting that PRL positivity alone may not independently predict SSA response. Some studies reported no significant differences in surgical remission rates between GH/PRL and GH-only adenomas [8, 20, 27], while others have suggested a less favorable prognosis for GH/PRL adenomas [3, 7]. The largest cohort, the ACRO-SPAIN study, which used a hybrid IHC and serum-based definition, did not show worse surgical remission outcomes in GH/PRL adenomas compared with GH-only adenomas [21]. However, they reported that biochemical control with preoperative SSA monotherapy was significantly poorer in GH/PRL tumors [21]. In their cohort, 18% were resistant to SSA, with predictors of non-response including Knosp grade > 2 (OR 8.75, *p* = 0.003), higher GH at diagnosis (OR 1.02, *p* = 0.031), and elevated postoperative GH levels (OR 1.05, *p* = 0.006) [28]. Importantly, in our cohort multimodal management resulted in long-term biochemical control in 87.2% of GH-only and 94.9% of GH/PRL patients at final follow-up, rates comparable to contemporary multimodal series [24, 25]. 

Beyond hormone co-expression, our data indicate that granulation pattern is a key determinant of tumor behavior. SGST consistently exhibited a more aggressive phenotype, with larger tumor size, higher rates of cavernous sinus invasion and optic chiasm compression, and markedly lower rates of both surgical remission and SSA responsiveness. These observations are consistent with recent literature showing that SGST are generally associated with larger and more invasive lesions, as well as poorer biochemical control and reduced responsiveness to first-generation somatostatin receptor ligands compared with DGST [6, 29]. 

Unlike prolactinomas, clear correlations have not been established between the size of GH-producing adenomas and serum GH levels [10, 22]. In our study, there was no significant correlation between tumor volume and baseline GH or IGF-1 in the full cohort, and only GH/PRL adenomas showed a positive correlation with PRL. These findings suggest that PRL may be a more reliable marker of tumor burden in GH/PRL adenomas.

This study has several limitations. Its retrospective, single-center design introduces potential selection bias and limits generalizability, although the uniformity of surgical expertise, perioperative decision-making, and postoperative management across the cohort represents a corresponding methodological strength. Although our sample size is comparable to many published series, it remains relatively modest for subgroup analyses, particularly for the small and heterogeneous “other” tumor group. T2-weighted MRI signal intensity was assessed qualitatively rather than by standardized quantitative measures, which may have introduced interobserver variability. Finally, SSTR2 and ERα immunohistochemistry were not available in our cohort, limiting mechanistic interpretation of SSA responsiveness and lineage-specific characterization of GH/PRL co-expressing subtypes.

In conclusion, although GH/PRL adenomas demonstrated higher proliferative activity, this did not translate into inferior surgical or medical outcomes compared with GH-only tumors. Treatment response was primarily determined by anatomical and pathological features rather than by hormone co-expression status alone. The observation that PRL correlated with tumor burden exclusively in GH/PRL adenomas further suggests subtype-specific biomarker utility.

These findings support individualized management strategies guided by morphological and radiological characteristics, and highlight the need for future studies incorporating transcription factor profiling and receptor expression analyses to refine prognostication in acromegaly.

## Data Availability

The dataset generated and analyzed during the current study contains patient names and national identification numbers and therefore cannot be publicly shared due to privacy and ethical restrictions. De-identified summary data may be made available from the corresponding author upon reasonable request.

## Declarations

### Prior presentation

This work has not been previously published or presented in any form.

## References

[1] Fleseriu M, Biller BMK, Freda PU, Gadelha MR, Giustina A, Katznelson L et al (2021) A Pituitary Society update to acromegaly management guidelines. Pituitary 24(1):1–13 33079318 10.1007/s11102-020-01091-7 PMC7864830

[2] Lv L, Jiang Y, Yin S, Hu Y, Chen C, Ma W et al (2019) Mammosomatotroph and mixed somatotroph-lactotroph adenoma in acromegaly: a retrospective study with long-term follow-up. Endocrine 66(2):310–8 31368083 10.1007/s12020-019-02029-1

[3] Rick J, Jahangiri A, Flanigan PM, Chandra A, Kunwar S, Blevins L et al (2019) Growth hormone and prolactin-staining tumors causing acromegaly: a retrospective review of clinical presentations and surgical outcomes. J Neurosurg 131(1):147–153 30215558 10.3171/2018.4.JNS18230

[4] Melmed S, Casanueva FF, Hoffman AR, Kleinberg DL, Montori VM, Schlechte JA et al (2011) Diagnosis and treatment of hyperprolactinemia: an Endocrine Society clinical practice guideline. J Clin Endocrinol Metab 96(2):273–288 21296991 10.1210/jc.2010-1692

[5] Coopmans EC, Korevaar TIM, van Meyel SWF, Daly AF, Chanson P, Brue T et al (2020) Multivariable Prediction Model for Biochemical Response to First-Generation Somatostatin Receptor Ligands in Acromegaly. J Clin Endocrinol Metab. ;105(9) 10.1210/clinem/dgaa387 32589751

[6] Durmuş ET, Atmaca A, Kefeli M, Çalışkan S, Mete O, Aslan K et al (2022) Age, GH/IGF-1 levels, tumor volume, T2 hypointensity, and tumor subtype rather than proliferation and invasion are all reliable predictors of biochemical response to somatostatin analogue therapy in patients with acromegaly: a clinicopathological study. Growth Horm IGF Res 67:101502 36115256 10.1016/j.ghir.2022.101502

[7] Guo X, Zhang R, Zhang D, Wang Z, Gao L, Yao Y et al (2021) Hyperprolactinemia and Hypopituitarism in Acromegaly and Effect of Pituitary Surgery: Long-Term Follow-up on 529 Patients. Front Endocrinol (Lausanne) 12:807054 35154007 10.3389/fendo.2021.807054 PMC8825499

[8] Wang M, Mou C, Jiang M, Han L, Fan S, Huan C et al (2012) The characteristics of acromegalic patients with hyperprolactinemia and the differences in patients with merely GH-secreting adenomas: clinical analysis of 279 cases. Eur J Endocrinol 166(5):797–802 22334636 10.1530/EJE-11-1119

[9] Araujo-Castro M, Marazuela M, Puig-Domingo M, Biagetti B (2023) Prolactin and Growth Hormone Signaling and Interlink Focused on the Mammosomatotroph Paradigm: A Comprehensive Review of the Literature. Int J Mol Sci. ;24(18) 10.3390/ijms241814002 PMC10531307 37762304

[10] Giustina A, Biermasz N, Casanueva FF, Fleseriu M, Mortini P, Strasburger C et al (2024) Consensus on criteria for acromegaly diagnosis and remission. Pituitary 27(1):7–22 37923946 10.1007/s11102-023-01360-1 PMC10837217

[11] Melmed S, di Filippo L, Fleseriu M, Mercado M, Karavitaki N, Gurnell M et al (2025) Consensus on acromegaly therapeutic outcomes: an update. Nat Rev Endocrinol 21(11):718–737 40804505 10.1038/s41574-025-01148-2

[12] Agrawal N, Ioachimescu AG (2020) Prognostic factors of biochemical remission after transsphenoidal surgery for acromegaly: a structured review. Pituitary 23(5):582–594 32602066 10.1007/s11102-020-01063-x

[13] Villa C, Vasiljevic A, Jaffrain-Rea ML, Ansorge O, Asioli S, Barresi V et al (2019) A standardised diagnostic approach to pituitary neuroendocrine tumours (PitNETs): a European Pituitary Pathology Group (EPPG) proposal. Virchows Arch 475(6):687–692 31578606 10.1007/s00428-019-02655-0

[14] Asa SLOR, Mete O (2025) Pituitary tumors. In: WHO Classification of Tumours Editorial Board. Endocrine and Neuroendocrine Tumours. Lyon: IARC; (WHO Classification of Tumours, 5th Edition, Vol 10). Available from: 2025 [Available from: https://tumourclassification.iarc.who.int/chapters/53

[15] Knosp E, Steiner E, Kitz K, Matula C (1993) Pituitary adenomas with invasion of the cavernous sinus space: a magnetic resonance imaging classification compared with surgical findings. Neurosurgery 33(4):610–617 discussion 7–8 8232800 10.1227/00006123-199310000-00008

[16] Lundin P, Pedersen F (1992) Volume of pituitary macroadenomas: assessment by MRI. J Comput Assist Tomogr 16(4):519–528 1629407 10.1097/00004728-199207000-00004

[17] Asa SL, Mete O, Perry A, Osamura RY (2022) Overview of the 2022 WHO classification of pituitary tumors. Endocr Pathol 33(1):6–26 35291028 10.1007/s12022-022-09703-7

[18] Wildemberg LE, Gadelha MR (2024) GH and prolactin co-secreting adenomas: It is time for a definition. J Clin Endocrinol Metab 110(1):e192–e3 38625822 10.1210/clinem/dgae262

[19] Chong L, Lou Y, Chen X, Zhao W, Zhang W, Zhang Z et al (2025) Comparison of the clinical and prognostic characteristics of patients with different pathological types in acromegaly. Front Endocrinol (Lausanne) 16:1571598 40421250 10.3389/fendo.2025.1571598 PMC12104045

[20] Dehghani M, Davoodi Z, Bidari F, Moghaddam AM, Khalili D, Bahrami-Motlagh H et al (2021) Association of different pathologic subtypes of growth hormone producing pituitary adenoma and remission in acromegaly patients: a retrospective cohort study. BMC Endocr Disord 21(1):186 34530798 10.1186/s12902-021-00850-2 PMC8447747

[21] Araujo-Castro M, Biagetti B, Menéndez Torre E, Novoa-Testa I, Cordido F, Pascual Corrales E et al (2024) Differences Between GH- and PRL-Cosecreting and GH-Secreting Pituitary Adenomas: a Series of 604 Cases. J Clin Endocrinol Metab 109(12):e2178–e87 38436926 10.1210/clinem/dgae126

[22] Petersenn S, Fleseriu M, Casanueva FF, Giustina A, Biermasz N, Biller BMK et al (2023) Diagnosis and management of prolactin-secreting pituitary adenomas: a Pituitary Society international Consensus Statement. Nat Rev Endocrinol 19(12):722–740 37670148 10.1038/s41574-023-00886-5

[23] Van Laethem D, Michotte A, Cools W, Velkeniers B, Unuane D, Andreescu CE et al (2020) Hyperprolactinemia in acromegaly is related to prolactin secretion by somatolactotroph tumours. Horm Metab Res 52(9):647–53 32757187 10.1055/a-1207-1132

[24] Guo X, Zhang R, Zhang D, Wang Z, Gao L, Yao Y et al (2022) Determinants of immediate and long-term remission after initial transsphenoidal surgery for acromegaly and outcome patterns during follow-up: a longitudinal study on 659 patients. J Neurosurg 137(3):618–628 35171834 10.3171/2021.11.JNS212137

[25] Yao S, Chen WL, Tavakol S, Akter F, Catalino MP, Guo X et al (2021) Predictors of postoperative biochemical remission in acromegaly. J Neurooncol 151(2):313–24 33394265 10.1007/s11060-020-03669-4 PMC10077515

[26] Starnoni D, Daniel RT, Marino L, Pitteloud N, Levivier M, Messerer M (2016) Surgical treatment of acromegaly according to the 2010 remission criteria: systematic review and meta-analysis. Acta Neurochir (Wien) 158(11):2109–2121 27586125 10.1007/s00701-016-2903-4

[27] Varlamov EV, Wood MD, Netto JP, Thiessen J, Kim J, Lim DST et al (2020) Cystic appearance on magnetic resonance imaging in bihormonal growth hormone and prolactin tumors in acromegaly. Pituitary 23(6):672–80 32870441 10.1007/s11102-020-01075-7

[28] Araujo-Castro M, Biagetti B, Menéndez E, Novoa-Testa I, Cordido F, Rodríguez Berrocal V et al (2025) Predictors of therapeutic failure in GH and prolactin co-secreting pituitary adenomas. Endocr Connect. ;14(7) 10.1530/EC-25-0103 PMC12268987 40590355

[29] Vuong HG, Dunn IF (2023) Clinical and prognostic significance of granulation patterns in somatotroph adenomas/tumors of the pituitary: a meta-analysis. Pituitary 26(6):653–659 37735314 10.1007/s11102-023-01353-0

